# Immunohistochemical study of the bcl‐2 in the pyramidal layer of the hippocampus in rats in hypertensive encephalopathy modeling

**DOI:** 10.1002/alz.088272

**Published:** 2025-01-09

**Authors:** Dmitry Mednikov, Alexey Vladimirovich Smirnov, Ivan Nikolaevich Tyurenkov

**Affiliations:** ^1^ Volgograd State Medical University, Volgograd Russian Federation; ^2^ Volgograd Medical Research Center, Volgograd Russian Federation

## Abstract

**Background:**

Closely related to the subfamily of proapoptotic proteins is the antiapoptotic protein bcl‐2, which acts as an intracellular blocker of the mitochondrial apoptotic pathway. By inhibiting the action of effector caspases, as well as blocking the release of AIF and cytochrome C, Bcl‐2 prevents regulated cell death and ensures survival in conditions of damage.

**Method:**

The study was performed on Wistar rats, which were subjected to gravitational overloads (9g) in the caudocranial vector for 5 minutes twice a day for 28 days.

**Result:**

An immunohistochemical study using antibodies to bcl‐2 in a group of rats with simulated hypertensive encephalopathy revealed a heterogeneous positive cytoplasmic reaction, mostly moderately expressed. In individual pyramidal neurons and glial cells, expression was mosaic in nature, where both dusty accumulation of IRM of weak intensity and deposits of more pronounced intensity were detected. Visually more intense staining was detected in neurons with deformed perikarya against the background of pronounced edema. Increased expression of bcl‐2 was also manifested in an increase in the relative area of the IRM. In CA1 of rats of the study group, this the indicator increased by 3.9% compared to the control (p<0.01). The most significant increase in the relative area of bcl‐2‐positive material was detected in CA2 and CA4, increasing, respectively, compared to the control by 5.6% (p<0.01) and 5.7% (p<0.01).

**Conclusion:**

Higher activity of bcl‐2 in the CA1 and CA3 zones may be one of the factors for the greater stability of CA2 and CA4 in relation to CA1 and CA3.

